# Pain Assessment of Elderly Patients with Cognitive Impairment in the Emergency Department: Implications for Pain Management—A Narrative Review of Current Practices

**DOI:** 10.3390/pharmacy5020030

**Published:** 2017-06-01

**Authors:** Joshua Jones, Tin Fei Sim, Jeff Hughes

**Affiliations:** School of Pharmacy, Curtin University, Western Australia 6102, Australia; Joshua.S.Jones@student.curtin.edu.au (J.J.); T.Sim@curtin.edu.au (T.F.S.)

**Keywords:** pain assessment, cognitive impairment, emergency department, elderly, falls, analgesic, quality use of medicines

## Abstract

Elderly people are susceptible to both falls and cognitive impairment making them a particularly vulnerable group of patients when it comes to pain assessment and management in the emergency department (ED). Pain assessment is often difficult in patients who present to the ED with a cognitive impairment as they are frequently unable to self-report their level of pain, which can have a negative impact on pain management. This paper aims to review how cognitive impairment influences pain assessment in elderly adults who present to the ED with an injury due to a fall. A literature search of EMBASE, ProQuest, PubMed, Science Direct, SciFinder and the Curtin University Library database was conducted using keyword searches to generate lists of articles which were then screened for relevance by title and then abstract to give a final list of articles for full-text review. Further articles were identified by snowballing from the reference lists of the full-text articles. The literature reports that ED staff commonly use visual or verbal analogue scales to assess pain, but resort to their own intuition or physiological parameters rather than using standardised observational pain assessment tools when self-report of pain is not attainable due to cognitive impairment. While studies have found that the use of pain assessment tools improves the recognition and management of pain, pain scores are often not recorded for elderly patients with a cognitive impairment in the ED, leading to poorer pain management in this patient group in terms of time to analgesic administration and the use of strong opioids. All healthcare professionals involved in the care of such patients, including pharmacists, need to be aware of this and strive to ensure analgesic use is guided by appropriate and accurate pain assessment in the ED.

## 1. Introduction

Pain assessment is the cornerstone of appropriate use of analgesics and the management of pain. It involves screening patients for pain, using an appropriate and validated assessment tool to assign a score that describes the severity and sometimes the nature of the pain, and then using the score in conjunction with guidelines, such as the World Health Organization analgesic ladder, to determine a suitable pain intervention for the patient [1,2]. Following the initial assessment, further periodic pain assessments are key in monitoring the efficacy of the chosen therapy and guiding dose adjustments and changes in pharmacotherapy to ensure that the patient is comfortable and that medicines are used appropriately [1]. As pharmacists are tasked with the responsibility of ensuring that patients achieve the best possible outcomes from their drug therapy, whilst at the same time ensuring quality use of medicines, they have a vested interest in confirming that pain assessments are timely and accurate. No more so than in vulnerable populations such as the elderly with cognitive impairment which limits their ability to self-report pain [3,4,5,6,7]. In such cases, pain often goes under-detected and under-managed despite patients having conditions/injuries known to cause pain, for example falls and fractures.

Injuries due to falls are a significant cause of morbidity and mortality worldwide [8]. Each year, approximately 28–35% of elderly adults (65 years of age and over) will experience a fall [9]. Falls contribute a significant economic burden for healthcare systems [9,10] and place sufferers at risk of disability, loss of function and reduced quality of life [8,11]. In elderly adults, injuries to the hip and thigh are the most common injury sustained from a fall, about 27% of all injuries, followed by injuries to the head (20.5%) [12]. About one in five injuries involve a fracture, with fractures to the neck of the femur accounting for 74% of the injuries to the hip or thigh [12].

Fractures are inherently painful and have been associated with an increased risk of developing an acute cognitive impairment such as delirium during a medical admission [13]. Furthermore, pain is associated with the development of delirium for patients admitted to hospital [14,15]. As cognitive impairment already increases the risk of injury due to a fall almost three-fold [16], as well as the risk of developing delirium during an admission [13,17,18], elderly patients presenting with falls often have a co-existing cognitive impairment. The presence of a neurocognitive disorder such as dementia is reported to be present in 10–30% of elderly patients in the emergency department (ED) [17,19,20]. This poses issues to their pain management if they are unable to self-report that they are in pain.

A regular and consistent approach to pain assessment is recognised as an important aspect of patient care as it informs and guides the management of pain and evaluation of the efficacy of pain interventions [21,22]. Self-report of pain is the preferred method of assessment, however this often becomes unattainable in elderly patients with cognitive impairment due to difficulties in comprehending commonly used pain assessment tools [3,4,5,6,7]. For example, a study by Lukas et al. [4] found that while the numerical rating scale (NRS) could be used by 75% of elderly adults with mild cognitive impairment to report pain, only 57% of patients with a moderate impairment and none of the severely impaired patients could utilise the tool. For patients with a cognitive impairment, alternative pain assessment methods, such as the observational and behavioural scales, may be more effective for assessing pain [22]. However, the extent of utilisation of such tools in the ED may be reduced due to challenges unique to this setting, such as time pressures and a lack of familiarity with the patient and therefore difficulty differentiating pain behaviours from the patient’s usual behaviour. Relatives and carers are often present in the ED and can provide an understanding of the patient’s usual behaviours and therefore allow identification of changes in behaviours related to pain [23,24,25]. Even so, caution is required when using observational scales and checklists to assess pain severity as the expression of pain behaviours varies between individuals who are experiencing equal levels of pain [26,27]. Furthermore, there is evidence to suggest that when a proxy is used to assess pain they tend to under report pain intensity when compared a patient’s self-reported pain score, which further complicates pain assessment [28,29,30].

Without an accurate pain assessment to guide appropriate pain management and to evaluate the efficacy of prescribed interventions, pain may be inadequately managed and patient suffering prolonged. The use of pain assessment tools and their value in guiding pain management is well documented in the literature, however there is limited information regarding the impact of cognitive impairment on pain assessment practices specifically in the ED setting. Therefore, this paper aims to review the current pain assessment practices and how the presence of cognitive impairment influences an elderly patient’s pain assessment in the ED when they present with an injury due to a fall.

## 2. Methods

**Figure 1:**
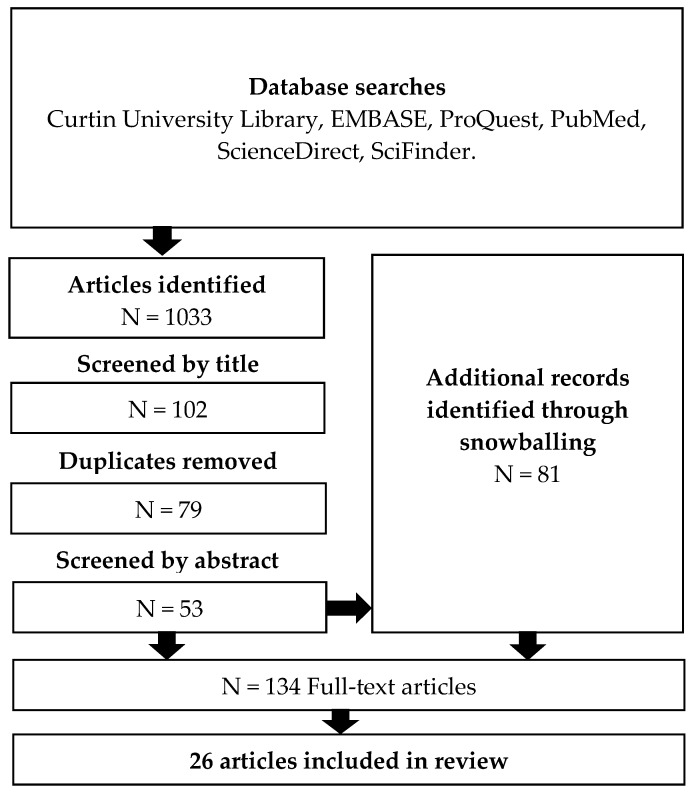
Literature search. Keyword searches of the databases were used to identify articles for potential inclusion in the review. Additional articles were identified through snowballing.

Articles were identified using keyword database searches and then snowballing from the reference lists of the relevant articles identified. The databases EMBASE, ProQuest, PubMed, Science Direct, SciFinder and the Curtin University Library database were searched using the terms ’emergency’, ’emergency department’, ‘hospital’, ’cognitive impairment’, ‘pain assessment’, ‘pain assessment methods’, ’elderly’, and ’frequency of pain assessment’. Searches were limited to peer-reviewed studies and articles that were published in English. If the search yielded an excessive number of results, then the search was restricted to articles published in the last ten years, however, one EMBASE search was limited to articles published within the last year, and another EMBASE search was limited to articles published within the last two years to reduce the number of results to less than 2000. In the three cases where the number of results was still greater than 1000, theme and relevance filters were applied to the results to reduce the number to a more manageable list. The 1033 articles identified in the searches were screened for relevance by title, reducing the list to 102 articles. After removing 23 duplicates, the remaining 79 articles were screened by abstract leaving a final list of 53 articles. Reference lists of these articles were used to identify a further 76 relevant articles. Manual searches added a further five articles to the final list of 134 articles for full-text review. Of these, 26 articles have been included in the review, selected based on their relevance to the aim of this paper (See Figure 1). Reasons for exclusion included lack of relevance, lack of focus on cognitively impaired patients and/or the elderly, wrong setting, lack of originality, poor methodology, and full version of the article was unavailable. One reviewer (JJ) conducted the literature searches to produce the list of 102 articles which was then screened by JH. The articles for full-text review were reviewed by JJ and screened by JH and TFS to give the final list of articles for inclusion in the review.

## 3. Findings

### 3.1. Methods of Pain Assessment

The use of standardised tools to assess pain improves the recognition of pain [31]. In an acute care setting, ideal pain assessment tools are easy to administer, rapid, accurate, valid, reliable, widely applicable to different patient groups, use self-report (if possible), and do not require special equipment or resources [32]. Numerous tools for pain assessment were identified in the literature and these can be categorised into self-report or observational pain assessment tools, as well as unidimensional or multidimensional tools. A systematic review conducted by Lichtner et al. [33] evaluated the psychometric properties and clinical utility of 28 pain assessment tools for use in people with dementia and concluded that while the Abbey Pain Scale, DS-DAT, DOLOPLUS-2, PACSLAC, PAINAD, Mahoney Pain Scale and ECPA have been identified as possible candidates for recommendation for common use, based on current evidence no definitive recommendations can be made.

Recent studies have found that ED staff tend to use visual or verbal analogue scales to assess pain [34,35,36]. One of these studies reported that there was no evidence that ED staff used pain assessment tools designed specifically for use in cognitively impaired people [34]. Instead, ED nurses tend to resort to their own intuition or observations of changes of physiological parameters, such as respiratory rate, instead of standardised observational pain assessment tools to determine pain levels [35]. This is not ideal as physiological parameters are not able to discriminate pain from other sources of distress and their absence does not reliably exclude the possibility that the individual is in pain [37,38]. The studies that were identified in the literature searches only report qualitative data regarding the utilisation of pain assessment tools in the ED for elderly patients with cognitive impairments. Therefore, future studies should attempt to describe pain assessment practices in the ED by quantifying the frequency of use of specific pain assessment tools in this group.

### 3.2. Frequency of Pain Assessment

Regular assessment of pain should be a part of any health care environment and is important for the delivery of quality care in the ED [21,22]. Despite this, studies show that pain is often under-assessed in the ED [34,36,39,40]. A study conducted in the United States (US) involving a retrospective review of medical charts of elderly patients with hip fractures in the ED found that whilst most patients had some record or mention of pain on their chart, 34% of patients’ pain was not assessed using a standardised method such as a NRS [40]. Similarly, Australian studies have found that of patients with a fractured neck of femur who present to the ED, between 32% and 47% of patients do not have a pain score recorded during their ED admission [34,36]. A summary of selected studies that investigate pain assessment practices in the ED can be found in Table 1. These studies suggest that both in Australia and the UK, pain tends to be under-assessed for elderly patients in the ED.

For patients with a cognitive impairment, the literature suggests that the frequency of pain assessment is likely to be even lower. A study conducted in the United Kingdom found that pain scores are not recorded in the ED for 55% of cognitively impaired patients compared with 25% of those cognitively intact [41]. No comparable Australian studies were identified in the literature that investigated the frequency of pain assessment in elderly patients with cognitive impairment in the ED.

### 3.3. Analgesia is Delayed for Elderly Patients with Cognitive Impairment

The delay to receiving analgesia when presenting to the ED with a painful condition is greater for elderly patients than for younger patients [36,42,43,44]. This delay is even greater for elderly patients with a cognitive impairment than for those without [34,45]. An Australian study by Fry et al. [34] found that elderly patients who are cognitively impaired who present to the ED with a long bone fracture waited, on average, 149 min to receive analgesia compared with just 72 min for cognitively intact patients. This is despite evidence to suggest that cognitively impaired patients do not perceive less pain than cognitively intact patients [7,46,47]. There is also evidence that patients who have a cognitive impairment are less likely to receive opioid analgesics compared to cognitively intact patients. A systematic review by Moschinski et al. [48] reported that eight of the 17 studies included in the review found that people with dementia receive less opioid analgesics than patients without dementia, but not less non-opioid analgesics, possibly due to increased concern about adverse effects such as sedation. Furthermore, when an opioid is prescribed to a person with cognitive impairment to manage pain associated with a hip fracture, it is less likely to be a strong opioid [41]. Improving pain assessment practices in the ED has the potential to reduce this delay and therefore improve pain management and patient outcomes [39].

### 3.4. Pain Assessment Improves Pain Management

The routine use of standardised pain assessment tools in the ED is important as use of such tools has been shown to increase the recognition and treatment of pain [31,49,50,51]. A cross-sectional study conducted in the US found that for older patients who presented to the ED with a diagnosed painful condition, the odds of receiving a prescription for an analgesic were 1.61 (95% CI: 1.42 to 1.82) if a pain score was documented compared with no pain score, and the prescription was more likely to be an opioid [50]. Another US study assessed the influence of the introduction of mandatory verbal numeric pain score assessments on analgesic use for patients presenting to the ED with typically painful conditions [39]. After the introduction of mandatory pain assessments, the number of patients receiving analgesics increased from 25% to 36% (*p* < 0.001), and the average wait time to receive analgesia was reduced by 39 min (95% CI: −7 to 84 min) [39]. These US studies demonstrate how increased utilisation of pain assessment tools has the potential to improve pain management. No studies were identified in the literature that report on the influence of pain assessment tool utilisation on pain management practices for cognitively impaired older patients in EDs in other countries.

### 3.5. Consequences of Poor Pain Management

Inadequate treatment of pain leads to immediate and delayed negative consequences for the patient. As well as discomfort, immediate consequences of untreated pain include physiological changes such as tachycardia, increased blood pressure, increased myocardial oxygen demand, changes of immune function, hypercoagulability, and metabolic changes [52]. Untreated pain also contributes to behavioural changes in elderly patients. A study conducted by Erel et al. [53] found that behavioural disorders, mainly aggressive behaviour, occurred in about one third of the elderly patients who reported pain as opposed to none of the patients who were pain free. Behavioural disorders were also more likely in patients who were both in pain and cognitively impaired (36.4%) than those who were in pain but did not have a cognitive impairment (16.7%) [53].

## 4. Conclusions

Elderly people are at risk of both injuries due to falls and cognitive impairment and therefore make up a large portion of presentations to the ED. Pain assessment in this patient group can be challenging as cognitive impairment reduces their ability to self-report pain. International studies show that the routine utilisation of standardised pain assessment tools can increase analgesic prescribing and potentially reduce wait time to analgesia in the ED. Studies were identified in the literature that presented data on the pain assessment practices of Australian, UK and US ED staff regarding cognitively impaired elderly patients, however no study could be identified that quantified the frequency of utilisation of specific pain assessment tools. There remains a problem of underutilisation of appropriate pain assessment tools in this patient group in the ED setting, with evidence to suggest that this is leading to suboptimal use of analgesics and poorer control of pain. Health professionals, including pharmacists, can play a role in promoting the regular use of standardised pain assessment tools in their patients to ensure that analgesics are used appropriately and are efficacious. Future studies should attempt to quantify the frequency of utilisation of various pain assessment tools in the ED focusing on elderly patients with cognitive impairments. A better understanding of current pain assessment practices would help to inform improvements in pain assessment and management for this vulnerable patient group.

## Figures and Tables

**Table 1:** Summary of selected studies that investigate pain assessment practices in the ED.

Authors	Design	Size	Setting	Findings
**Fry, Chenoweth, and Arendts (2016) [35]**	Focus group interviews, qualitative	80 emergency nurses, 16 focus groups	Four Australian EDs	Nurses reported that visual or verbal analogue scales for pain assessment are often unsuitable in cognitively impaired patients. When these methods failed, nurses relied on clinical judgement and physiological measures (e.g., respiratory rate) rather than standardised observational pain assessment tools.
**Fry, Arendts, Chenoweth, and MacGregor (2015) [34]**	Retrospective cross-sectional study of patient ED records	255 elderly patients with long bone fractures	Four Australian EDs	Only 68% of patients had a pain score during their ED admission. The verbal analogue scale was routinely used. There was no evidence that ED staff used tools designed specifically for cognitively impaired people. 204 of 255 patients received analgesia in the ED. A cognitively impaired patient was not more likely to receive no analgesia compared with a cognitively intact patient. Median wait time to analgesia was 72 min for cognitively intact patients compared with 149 min for cognitively impaired patients.
**Holdgate, Shepherd, and Huckson (2010) [36]**	Retrospective cross-sectional study of patient ED records	646 patients with fractured neck of femur	36 EDs across 5 Australian states	Confusion/dementia was reported as a barrier to analgesia in 42 out of the 99 patients who had a barrier recorded. 47% of patients had no documented pain score during their ED admission. Visual analogue scales, verbal numerical pain scores and Likert scales were used.
**McDermott, Nichols, and Lovell (2014) [41]**	Retrospective cross-sectional study of patient ED records	224 patients with fractured neck of femur	Wythenshawe Hospital ED, Manchester, United Kingdom.	A pain score was documented for 45% of cognitively impaired patients compared with 75% of cognitively intact patients. 45% of cognitively impaired patients were not offered an analgesic while only eight percent of cognitively intact patients had no prescribed analgesia.
